# A New Motor for the Dental Profession

**Published:** 1887-03

**Authors:** 


### A New Motor for the Dental Profession.

An improved electric motor has been devised and made by the Detroit Motor Co. which gives indications of being a valuable appliance for the dentist.

It supercedes the ordinary dental engine, has far greater velocity, and ample power for any operation in the mouth in which an engine is employed. The engine with its switch board and attachments are well nigh perfect. The power, of course, is derived from a battery, and one has been secured for this motor that seems to possess almost every reasonable quality. It consists of six cells or jars, 6x8, placed in a box. The battery needs but little attention. It has two fluids, one of which requires to be changed once for every 75 to 100 hours of actual work; the other will give from 300 to 400 hours of efficient work without change or addition. Except when necessary to change the fluid, the battery must not be touched.

Two or three cells give ample power to run the dental mallet and the electric mouth-lamp as well.

The cost per hour for actual work is less than one cent, as there is practically no consumption of material when the battery is at rest. A gentleman who has quite a large practice has had this motor and battery in constant use for over three months. The battery has required only a few minutes attention during that time. Probably less time and attention was given it, than would be required by an ordinary dental engine. We have had this motor in use for about two weeks, and thus far it has rendered entire satisfaction, and the feeling is that a return to the ordinary dental engine would be a backward movement.

